# BioForge: a fast and safe macromolecular structure preparation library in Rust

**DOI:** 10.1093/bioinformatics/btag616

**Published:** 2026-10-07

**Authors:** Tony Kan, Ted Yu, Soo-Kyung Kim, Gaoyuan Liu, Yixin C Xu, Sejun Kim, William A Goddard

**Affiliations:** Materials and Process Simulation Center, Beckman Institute, California Institute of Technology, Pasadena, CA 91125, United States; Materials and Process Simulation Center, Beckman Institute, California Institute of Technology, Pasadena, CA 91125, United States; Materials and Process Simulation Center, Beckman Institute, California Institute of Technology, Pasadena, CA 91125, United States; Materials and Process Simulation Center, Beckman Institute, California Institute of Technology, Pasadena, CA 91125, United States; Materials and Process Simulation Center, Beckman Institute, California Institute of Technology, Pasadena, CA 91125, United States; Materials and Process Simulation Center, Beckman Institute, California Institute of Technology, Pasadena, CA 91125, United States; Materials and Process Simulation Center, Beckman Institute, California Institute of Technology, Pasadena, CA 91125, United States

## Abstract

**Summary:**

BioForge is the first macromolecular structure preparation library for Rust, complementing sequence-analysis libraries such as Rust-Bio with cleaning, geometry-based missing-atom repair, protonation, and solvation as a force-field-agnostic preprocessing stage. Across 22 structures (1439–3 819 839 atoms) on a single CPU core, BioForge scales linearly in wall time (fitted $N^{0.89}$) versus super-linear $N^{1.17}$ for PDBFixer and $N^{1.25}$ for PDB2PQR, completing the 3.8M-atom 9MJN in 5.1 s at 1.1 GB peak (PDBFixer: error, 273 GB); PDB2PQR is limited to fewer than 100 000 atoms. BioForge also compiles to WebAssembly for browser deployment and supports direct embedding into simulation and parameterization pipelines via its application programming interface.

**Availability and implementation:**

An MIT-licensed Rust library crate (*bio-forge*) and a WebAssembly package (*bio-forge-wasm*) extend the Rust bioinformatics ecosystem with structure preparation, both published on https://crates.io and NPM respectively; the same WebAssembly binary powers a client-side web application at https://bio-forge.app that runs entirely in the browser without a server or data upload. Source code and precompiled command-line binaries are freely available at https://github.com/TKanX/bio-forge; an archival snapshot of the version used for all reported experiments is deposited at Zenodo (https://doi.org/10.5281/zenodo.21627953).

## 1 Introduction

Structures deposited in the Protein Data Bank lack hydrogen positions, may have heavy atoms unresolved, and carry heterogeneous atom nomenclature, yet force-field calculations require a canonical, fully-connected molecular graph: explicit hydrogens, assigned protonation states, and a bond topology. Structure preparation bridges this gap as the universal upstream step for any atomistic calculation.

The most widely used tools for this task are PDBFixer (Eastman 2025, https://github.com/openmm/pdbfixer) and PDB2PQR (Dolinsky *et al.* 2004), both implemented primarily in Python; interpreter overhead and Python’s memory model impose a practical ceiling on throughput and peak memory for large assemblies. PDBFixer additionally includes a post-placement energy-minimization pass via OpenMM (Eastman *et al.* 2017). PDB2PQR targets Poisson–Boltzmann electrostatics setup; it performs no size check and crashes on structures exceeding 99 999 atoms. Neither tool exposes a compiled-language application programming interface (API); both require a Python interpreter at the pipeline boundary.

Rust (Klabnik and Nichols 2023) has gained traction in scientific computing for its compile-time memory and data-race safety, and its bioinformatics and simulation ecosystem now encompasses sequence analysis (Köster 2016), molecular dynamics (MD) integration (O’Connor 2025, https://github.com/David-OConnor/dynamics), and MD trajectory analysis (Yesylevskyy 2025); DREID-Forge (Kan 2026a, https://github.com/caltechmsc/dreid-forge) and DREID-Pack (Kan 2026b, https://github.com/caltechmsc/dreid-pack) further apply DREIDING (Mayo *et al.* 1990) to force-field parameterization and protein side-chain packing as direct downstream consumers. No existing Rust crate addresses structure preparation, however, leaving simulation and parameterization pipelines to invoke Python at their upstream step. We present BioForge, the first macromolecular structure preparation library for Rust: a deliberately decoupled, force-field-agnostic first stage that produces a canonical molecular graph, with *(i)* five independently invokable pipeline stages (clean, repair, protonate, solvate, topology) and no embedded energy minimization, built on Kabsch singular value decomposition (SVD) template alignment (Kabsch 1976) and $pK_{a}$-threshold protonation; *(ii)* an O(1) spatial hash and data-parallel iterators via Rayon (Matsakis and Stone 2015, https://github.com/rayon-rs/rayon) for scalable throughput; and *(iii)* a WebAssembly (WebAssembly Community Group 2026, https://webassembly.github.io/spec/core/) build for browser deployment and an embeddable library API for integration into Rust simulation and design pipelines.

## 2 Implementation

BioForge exposes five pipeline stages—clean, repair, protonate, solvate, and topology—each accepting a mutable Structure and a typed configuration struct; stages share no state and may be invoked selectively or composed into a full pipeline (Fig. 1). Residue geometry templates covering all standard amino acids, nucleotides, and solvent molecules are derived from ideal coordinates in the wwPDB Chemical Component Dictionary (wwPDB Consortium 2026, https://www.wwpdb.org/data/ccd) and compiled into the binary at build time, eliminating any runtime file I/O for standard residues. A uniform spatial hash (O(N) construction, O(1) average-case neighbour query) and Rayon data-parallel iterators (Matsakis and Stone 2015) serve as shared infrastructure across geometry-intensive stages.

**Figure 1:**
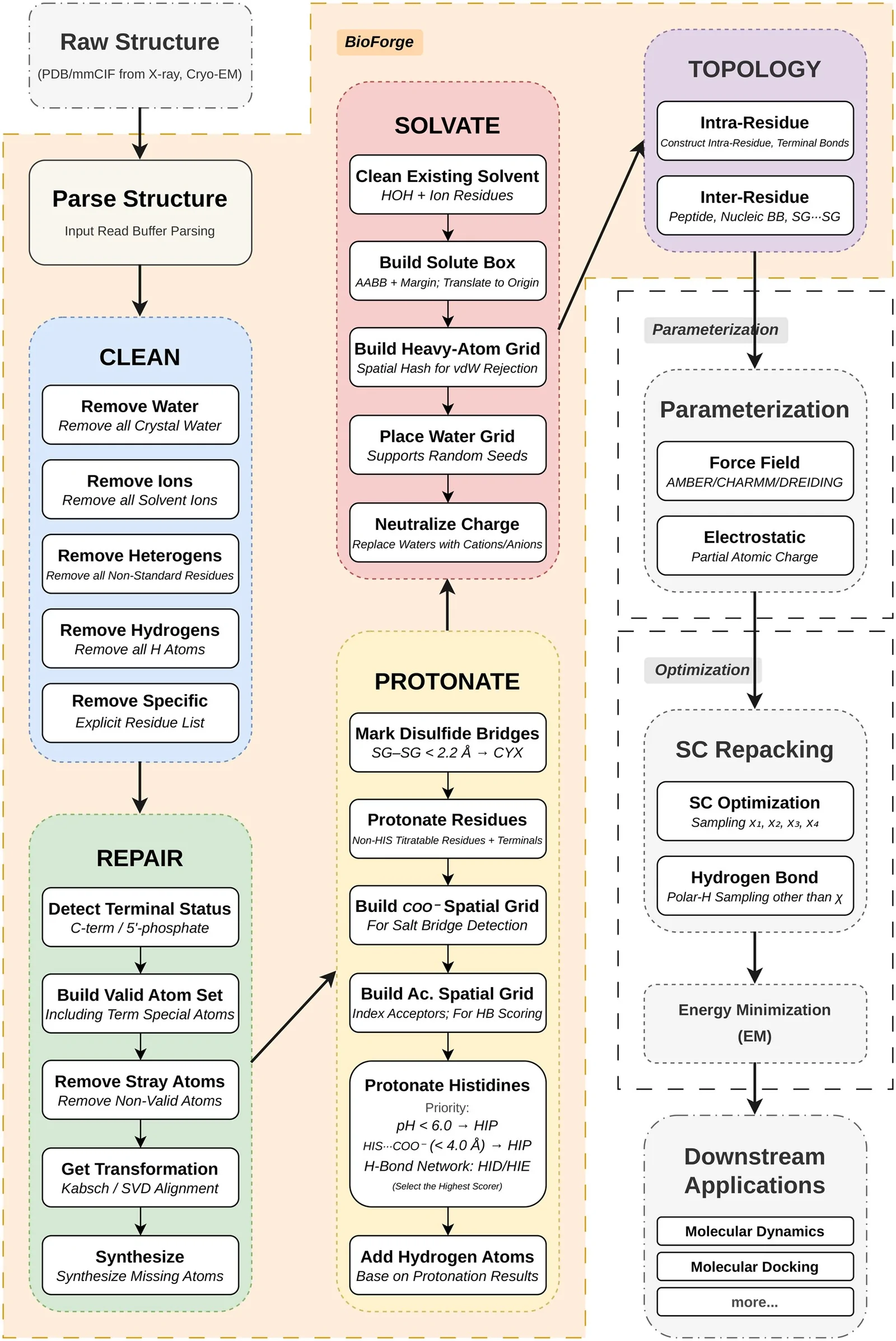
Overview of the BioForge structure preparation pipeline (see *Implementation* for details).

### 2.1 Cleaning

A single parallel pass removes any combination of water, ions, and heterogens—explicit keep and remove lists override these filters, in that order—and optionally strips all hydrogens; emptied chains are pruned.

### 2.2 Repair

For each standard residue the valid atom set is constructed from its residue template, extended with OXT at protein C-termini and OP3 at 5’-phosphorylated nucleic-acid termini. Atoms absent from this set are removed; remaining heavy atoms serve as alignment anchors. A single anchor produces a pure translation; two anchors yield an axis-angle rotation; three or more anchors are aligned by Kabsch SVD (Kabsch 1976)—3×3 cross-covariance $H=\sum_{i}v_{\text{obs},i}v_{\text{tmpl},i}^{⊤}$ of centroid-subtracted observed and template anchor positions, decomposed with a determinant sign correction for right-handedness—yielding a rotation R and the translation $t=c_{\text{obs}}-Rc_{\text{tmpl}}$ between anchor centroids. Missing heavy atoms are synthesized by applying (R,t) to their template positions; terminal atoms are placed instead from local bond geometry. All residues are dispatched independently across threads.

### 2.3 Protonation

The protonation stage assigns charge states and constructs hydrogen coordinates for the resolved states as two decoupled phases, both parallelized across residues; when no target pH is provided, existing protonation states are preserved and hydrogen coordinates are constructed directly, allowing residue-level control by the caller.

Disulfide detection precedes all titration: CYS pairs with SG⋯SG<2.2 Å (spatial-hash query) are relabeled CYX and excluded from subsequent modification. When a target pH is supplied, six titratable side chains and three terminal groups are assigned by commonly adopted $pK_{a}$ thresholds (ASP 3.9, GLU 4.2, LYS 10.5, ARG 12.5, CYS 8.3, TYR 10.0, N-terminal amine 8.0, C-terminal carboxylate 3.1, terminal phosphate 6.5; cf. (Nelson and Cox 2012)).

Histidine state requires special treatment because three forms coexist (HIP/HID/HIE) and the optimal tautomer depends on the local electrostatic environment. At pH < 6.0 all histidines are doubly protonated (HIP). Above this threshold, a salt-bridge screen checks whether ND1 or NE2 lies within 4.0 Å of any carboxylate oxygen, queried against a spatial grid of ASP${\;}^{-}$, GLU${\;}^{-}$, and C-terminal COO${\;}^{-}$ atoms; a match yields HIP. Remaining histidines enter a hydrogen-bond network evaluation: a hypothetical N–H direction is constructed from the bisector of the two flanking ring carbons, and acceptors (N, O, F) within 3.5 Å are scored as


(1) $$S=\sum_{A}\frac{{\;\text{cos}\;}^{2}\theta_{\text{DHA}}}{r_{\text{HA}}^{2}},\;r_{\text{HA}}<2.7\;Å,\;\;\text{cos}\;\theta_{\text{DHA}}>0;$$

$\theta_{\text{DHA}}$ is the donor–hydrogen–acceptor angle, $r_{\text{HA}}$ the hydrogen–acceptor distance; ${\;\text{cos}\;}^{2}\theta_{\text{DHA}}$ enforces directionality (zero at 90°, maximum at linearity) and $r_{\text{HA}}^{-2}$ provides a dipole-field distance decay; the tautomer (HID or HIE) with the higher score is selected.

Hydrogen coordinates are then placed from template geometry for all 29 protein protonation states and 10 nucleotide templates, with terminal-specific rules for protein N-/C-termini (including proline) and nucleic-acid 5′- and 3′-ends.

### 2.4 Solvation

An axis-aligned bounding box over all solute heavy atoms is expanded by a configurable margin (default 10 Å) to define an orthorhombic cell, and the solute is translated to the padded origin. Water molecules are placed on a regular grid at 3.1 Å spacing, with placement parallelized across z-slices; each candidate is rejected if any of its atoms falls within 2.4 Å of an existing heavy atom via the spatial hash. An optional random seed governs both the independent random Euler rotation applied to each accepted water and the subsequent ion placement, ensuring full reproducibility. The system charge is adjusted to a user-specified target (default: neutral) by replacing randomly selected waters with counterions drawn from a user-configured species set (monovalent Na${\;}^{+}$, K${\;}^{+}$, Li${\;}^{+}$, Cl${\;}^{-}$, Br${\;}^{-}$, I${\;}^{-}$, F${\;}^{-}$; divalent Mg${\;}^{2+}$, Ca${\;}^{2+}$, Zn${\;}^{2+}$).

### 2.5 Topology

The topology stage constructs the full covalent bond graph required by downstream force-field parameterization—where bond connectivity determines hybridization states and atom types (Mayo *et al.* 1990, Kan 2026a)—and by atomistic simulation engines that derive bond, angle, and dihedral terms from the graph. Intra-residue bonds are drawn from residue templates; inter-residue backbone bonds are inferred by distance (peptide C–N ≤ 1.5 Å, nucleic O3’–P ≤ 1.8 Å); disulfide bonds are detected via the spatial hash at a 2.2 Å SG–SG cutoff by default. Non-standard residues are handled through caller-supplied templates. The final bond list is sorted in parallel and deduplicated.

## 3 Usage

### 3.1 Command-line interface

The five pipeline stages are exposed as clean, repair, hydro (protonation and hydrogen placement), solvate, and topology; info and transform complement them without altering chemical composition. All seven operate as independent UNIX filters (stdin→stdout), so any subset composes via pipe without intermediate files; -i/-o redirect to file, with format (PDB or mmCIF) inferred from extension or set via—format/—out-format. info reports per-chain residue/atom counts, polymer type, unit-cell parameters, and estimated charge to stderr, passing the structure through as a transparent filter. clean selectively strips water, ions, hydrogens, or hetero residues (--water, --ions, --hydrogens, --hetero), with per-residue--keep/--remove overrides. hydro accepts a target pH (--ph), histidine tautomer strategy (--his: hid/hie/random/network), —no-strip to retain existing hydrogens, and--no-his-salt-bridge. solvate controls box margin (--margin, default 10 Å), grid spacing (--spacing, 3.1 Å), ion species (--cation: Na/K/Li/Mg/Ca/Zn; --anion: Cl/Br/I/F), charge target (--neutralize or--target-charge), and random seed (--seed). transform applies centering (--center, --center-mass), axis rotation (--rotate-{x, y, z}), and translation (--translate) in fixed order. topology emits CONECT (PDB) or _struct_conn (mmCIF) bond records, with configurable disulfide cutoff (--ss-cutoff, default 2.2 Å) and caller-supplied MOL2 ligand definitions (--hetero-template). The command-line interface (CLI) is installable via cargo install bio-forge or as a precompiled binary; the repository’s examples/directory gives worked invocations, including a full preparation run composed entirely through shell pipes.

### 3.2 Library API

io exposes PDB and mmCIF readers and writers, ops the five pipeline stages; the optional parallel Cargo feature gates Rayon, and disabling it yields a single-threaded build for environments without OS threading. Full API documentation is published at https://docs.rs/bio-forge.

### 3.3 WebAssembly

The *bio-forge-wasm* package on npm wraps the same pipeline via wasm-bindgen TypeScript bindings; the same binary powers https://bio-forge.app, running structure preparation client-side without data upload.

## 4 Results

BioForge 0.4.1, PDBFixer 1.12.0 (OpenMM 8.5.0), and PDB2PQR 3.7.1 (PROPKA 3.5.1, Python 3.13.12) were benchmarked on 22 PDB structures (Table S1, available as supplementary data at *Bioinformatics* online) selected to span N=1439–3 819 839 atoms and composed entirely of standard protein and nucleic-acid residues supported by all three tools (Fig. 2). All runs were single-threaded on an Intel Xeon Platinum 8352Y (2.20 GHz, 503 GB RAM) to isolate algorithmic efficiency; PDB2PQR provides no multi-threaded mode. Each configuration executed one warmup followed by five timed runs; the median wall-clock time and peak resident memory (VmHWM) are reported. File I/O was excluded for all three tools. BioForge cleans (removes water, ions, existing hydrogen, and heterogens), performs template-based missing-atom repair, and protonates at pH 7.4. PDBFixer performs the same logical steps but unconditionally invokes OpenMM energy minimization during both heavy-atom and hydrogen placement; this behaviour is intrinsic to PDBFixer’s design and cannot be disabled. Missing-residue insertion was excluded from all three configurations: BioForge and PDB2PQR do not implement it, and PDBFixer’s--add-residues is off by default. PDB2PQR additionally performs steric debumping, hydrogen-bond optimization, and AMBER force-field assignment at standard protonation states; structure-specific pKa prediction via PROPKA (Søndergaard *et al.* 2011) is not the CLI default (--titration-state-method defaults to None) and was not enabled here. PDB2PQR serializes CIF input as fixed-width PDB-format lines internally; values exceeding a column width (atom serial > 99 999, multi-character chain identifiers) produce malformed records that crash the downstream PDB parser with an uncaught ValueError (cif.py). This restricted the PDB2PQR benchmark to the 12 structures with ≤99 999 input atoms. PDBFixer accepts both PDB and mmCIF input but its CLI writes PDB output only.

**Figure 2:**
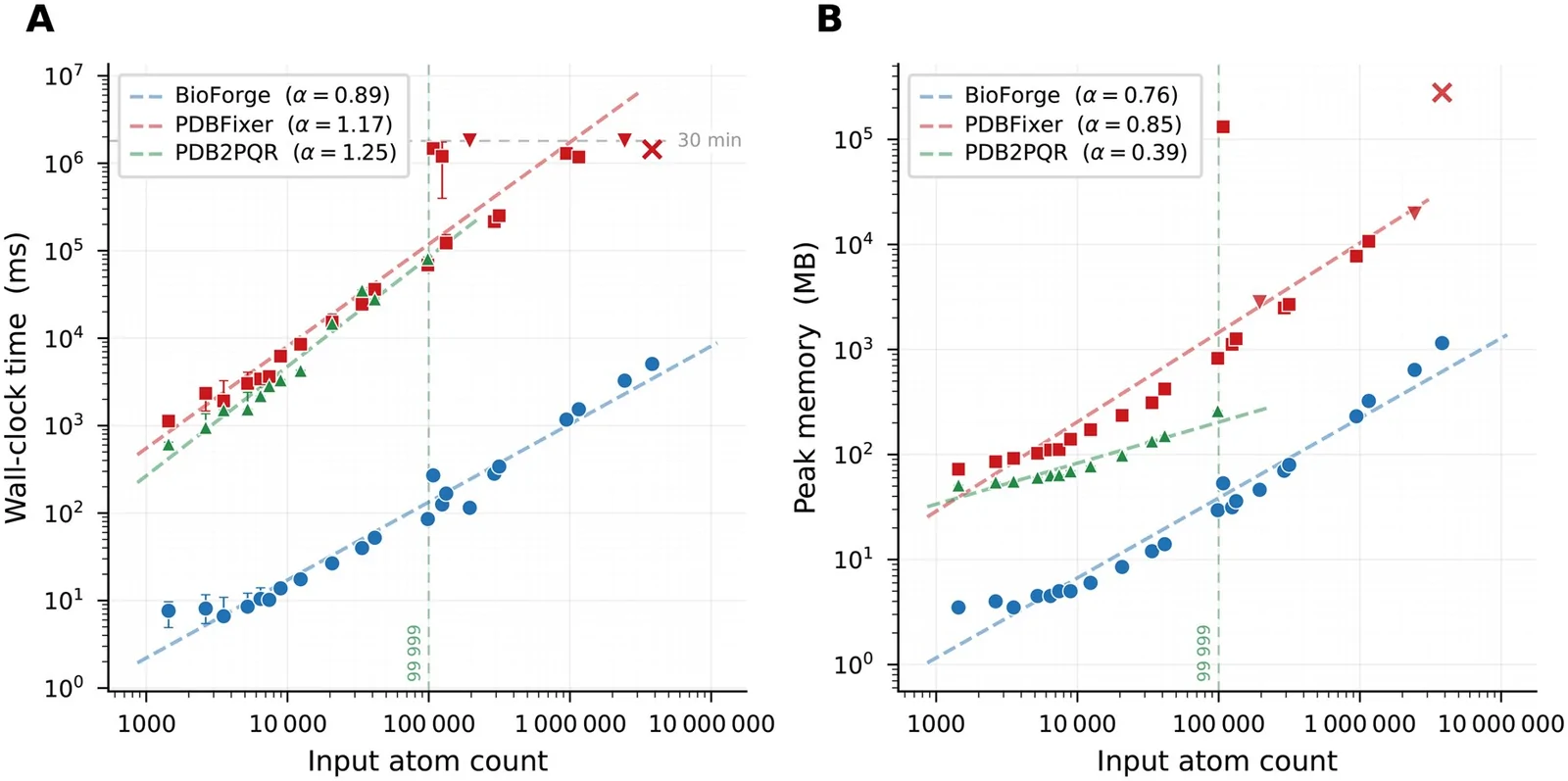
Performance scaling across 22 PDB structures (single-threaded). (A) Wall-clock time vs. input atom count. (B) Peak memory (VmHWM) vs. input atom count. Dashed lines: power-law fits ($t\propto N^{\alpha }$) for each tool. Inverted triangles (▼): timeout at 30 min; crosses (×): runtime error. Vertical dashed line: PDB2PQR 99 999-atom input limit.

Power-law fits ($t\propto N^{\alpha }$) on wall-clock time yield α=0.89 ($R^{2}=0.97$) for BioForge, 1.17 ($R^{2}=0.89$) for PDBFixer, and 1.25 ($R^{2}=0.97$) for PDB2PQR (Fig. 2A). Memory scaling exponents are 0.76 ($R^{2}=0.96$) and 0.85 ($R^{2}=0.70$) for BioForge and PDBFixer, respectively (Fig. 2B). For PDB2PQR the apparent memory exponent is 0.39; however, a linear model m=50MB+2.3KB · N fits the 12 data points with $R^{2}=0.994$ (vs. 0.92 for the power law). The 50 MB constant is confirmed by direct measurement: loading all benchmark dependencies (pdb2pqr, propka) with no structure processed yields VmHWM = 50.4 MB, matching the regression intercept to within 0.4%. The low apparent exponent is therefore an artifact of this size-independent baseline dominating a narrow dynamic range: total memory spans only 5.1× over a 68.5× range in *N*, while the data-proportional term scales linearly with *N*. On the 19 structures completed by both BioForge and PDBFixer, BioForge is a median 612× faster (range 148–9567×) and uses a median 28× less memory (range 21–2480×). BioForge completes the largest structure (PDB 9MJN; 3 819 839 atoms) in 5.1 s with 1.1 GB peak memory. PDBFixer times out at 30 min on PDB 3J6B (195 137 atoms) and PDB 3J3Q (2 440 800 atoms), and fails with a std::bad_alloc exception while allocating an OpenMM Context at 272.8 GB resident memory on 9MJN. PDB2PQR’s largest successful run is PDB 1JJ2 (98 543 atoms) at 80.8 s. Table S1, available as supplementary data at *Bioinformatics* online lists per-structure timings, peak memory, and failure modes for all three tools.

## 5 Discussion

BioForge’s cost is linear in system size by construction: each residue undergoes one closed-form Kabsch SVD alignment and one $pK_{a}$-threshold state assignment, both O(1) per residue with no inter-residue coupling. The fitted α=0.89 reflects a size-independent baseline inside the timed region—one-time deserialization of the compiled-in residue templates—and the fit over $N\geq 10^{4}$ gives 1.00 ($R^{2}=0.97$). PDBFixer’s super-linear exponent (α=1.17) and high memory variance ($R^{2}=0.70$) arise because its placement stages invoke OpenMM energy minimization, whose cost scales with the reconstructed atom count rather than the input. In a supplementary multithreaded benchmark (32 threads, loop modelling enabled), PDBFixer terminated with a segmentation fault on four of 22 structures (3J6B, 3J79, 3JC8, 4BTS) across all five timed runs each; the same structures completed without error under single-threaded execution and under BioForge with 32 threads.

BioForge produces a correct molecular graph—canonical heavy atoms, assigned protonation states, explicit bond topology—and stops there. Partial-charge assignment, atom typing, sidechain repacking, and energy minimization are outside its scope and are left to downstream tools, including DREID-Forge (Kan 2026a) for parameterization and DREID-Pack (Kan 2026b) for repacking, both of which consume BioForge output directly.

Atom repair operates within existing residues: missing heavy atoms are placed by Kabsch-guided template superposition; loop modelling is outside scope. Protonation states are assigned by fixed reference $pK_{a}$ thresholds, which approximate standard solution values; microenvironment shifts that displace a residue’s true $pK_{a}$ across the threshold—as predicted by tools such as PROPKA (Søndergaard *et al.* 2011)—are outside BioForge’s scope. Histidine tautomers are assigned by geometry-based hydrogen-bond scoring (1); applications requiring environment-corrected protonation should supply explicit state assignments via the per-residue variant API. Future work will extend the pipeline with missing-residue loop modelling, lipid-membrane construction for transmembrane proteins, and Python bindings via pyo3.

## Supplementary Material

btag616_Supplementary_Data

## Data Availability

An MIT-licensed Rust library crate (*bio-forge*) and a WebAssembly package (*bio-forge-wasm*) extend the Rust bioinformatics ecosystem with structure preparation, both published on https://crates.io and NPM respectively; the same WebAssembly binary powers a client-side web application at https://bio-forge.app that runs entirely in the browser without a server or data upload. Source code and precompiled command-line binaries are freely available at https://github.com/TKanX/bio-forge; an archival snapshot of the version used for all reported experiments is deposited at Zenodo (https://doi.org/10.5281/zenodo.21627953).
