# Supplementary material for: BioForge: a fast and safe macromolecular structure preparation library in Rust
Source: Bioinformatics. 2026 Oct 7;42(10):btag616. doi: 10.1093/bioinformatics/btag616 (PMC13657428; doi:10.1093/bioinformatics/btag616)
Supplement: Supplementary file 1 [file btag616_supplementary_data.pdf]

Supplementary Information

BioForge: A Fast and Safe Macromolecular Structure Preparation Library in Rust

Tony Kan, Ted Yu, Soo-Kyung Kim, Gaoyuan Liu, Yixin Clem Xu, Sejun Kim, William A. Goddard III

Contents

Table S1: Per-structure benchmark results

Table S2: BioForge pipeline stage breakdown

Table S1. Per-structure benchmark results on 22 RCSB PDB structures (single-threaded). Versions: BioForge 0.4.1, PDBFixer 1.12.0 (OpenMM 8.5.0), PDB2PQR 3.7.1 (PROPKA 3.5.1, Python 3.13.12). Hardware: Intel Xeon Platinum 8352Y (2.20 GHz, 503 GB RAM). N: input atom count; N_r: input residue count. $\tilde{t}$: median wall-clock time of five timed runs (warmup excluded from median). CoV: coefficient of variation (%) of BioForge $\tilde{t}$ across five timed runs; reported for BioForge only because PDBFixer and PDB2PQR run at second-to-minute timescales where scheduling noise is negligible relative to total time. Elevated CoV (>10 %) for small structures ($N < 10^4$, $\tilde{t} < 20$ ms) reflects OS-scheduling and cache noise at millisecond timescales and does not affect speedup conclusions. $\tilde{m}$: median peak resident memory (VmHWM) of five timed runs. All PDBFixer and PDB2PQR runs that were submitted either completed fully or failed completely; partial success never occurred. ρ_t, ρ_m: PDBFixer-to-BioForge ratio of $\tilde{t}$ and $\tilde{m}$. BioForge completed all 22 structures. † timeout (>30 min); ‡ runtime error (`std::bad_alloc` during OpenMM Context allocation at 272.8 GB resident memory on 9MJN). $\tilde{m}$ marked † or ‡: peak from 2 s-interval external memory monitor, not self-reported VmHWM. *n.r.*: not run (PDB2PQR 5-digit serial-number overflow for $N > 99\,999$).

			**BioForge**			**PDBFixer**		**PDB2PQR**		**BF vs. PF**	
**PDB**	N	N_r	$\tilde{t}$ (ms)	CoV (%)	$\tilde{m}$ (MB)	$\tilde{t}$ (s)	$\tilde{m}$ (MB)	$\tilde{t}$ (s)	$\tilde{m}$ (MB)	ρ_t	ρ_m
102L	1439	299	7.6	30.9	4	1.1	72	0.6	51	148	21
104L	2636	360	8.1	27.8	4	2.3	86	1.0	54	289	21
1A04	3535	771	6.6	27.3	4	1.9	92	1.5	56	290	26
11AS	5220	740	8.5	17.3	4	3.0	103	1.5	60	356	23
168L	6445	820	10	14.4	4	3.4	111	2.2	64	327	25
12E8	7429	1637	10	9.6	5	3.6	112	2.9	64	357	22
1A4Y	8939	1299	14	5.3	5	6.2	140	3.3	70	452	28
9OPY	12 388	1570	18	0.7	6	8.5	172	4.3	78	486	29
1E9R	20 718	3486	27	0.7	8	15.3	236	14.7	98	574	28
1A2V	33 726	6492	40	0.7	12	24.4	312	35.3	134	612	26
1E9S	41 513	6636	52	0.7	14	36.2	421	27.9	151	693	30
1JJ2	98 543	14 702	86	5.0	30	68.7	824	80.8	260	803	28
3JC8	107 640	26 910	270	0.3	53	1474	131 990	*n.r.*	—	5460	2480
3J79	124 514	9872	125	0.6	31	1200	1119	*n.r.*	—	9567	36
3J9G	132 930	16 710	167	1.0	36	122	1265	*n.r.*	—	732	35
3J6B	195 137	9558	115	1.4	46	†	2795†	*n.r.*	—	—	—
1VVJ	291 123	21 387	282	2.4	70	215	2486	*n.r.*	—	763	35
4BTS	315 512	29 904	341	1.3	80	252	2688	*n.r.*	—	739	34
7EZX	944 406	118 770	1173	0.9	231	1296	7736	*n.r.*	—	1105	33
6QVK	1 155 500	149 940	1539	0.4	324	1178	10 722	*n.r.*	—	766	33
3J3Q	2 440 800	313 236	3270	0.4	640	†	19 495†	*n.r.*	—	—	—
9MJN	3 819 839	494 049	5074	1.1	1150	‡	279 326‡	*n.r.*	—	—	—

Table S2. BioForge 0.4.1 pipeline stage breakdown (single-threaded, 22 structures). $\tilde{t}$: median of five timed runs (ms); f: fraction of total time (%). Stages are executed sequentially: CLEAN removes water, ions, existing hydrogen, and heterogens; REPAIR places missing heavy atoms via Kabsch/SVD template alignment; HYDRO assigns protonation states and places hydrogen at pH 7.4. All 22 structures completed without error.

		CLEAN		REPAIR		HYDRO		**total**
**PDB**	N	$\tilde{t}$ (ms)	f (%)	$\tilde{t}$ (ms)	f (%)	$\tilde{t}$ (ms)	f (%)	$\tilde{t}$ (ms)
102L	1439	0.018	0.2	5.40	70.7	2.22	29.1	7.6
104L	2636	0.014	0.2	5.03	62.0	3.16	38.9	8.1
1A04	3535	0.029	0.4	3.39	51.2	3.20	48.4	6.6
11AS	5220	0.015	0.2	3.69	43.3	4.82	56.5	8.5

Continued on next page

... continued

**PDB**	N	CLEAN $\tilde{t}$ (ms)	CLEAN f (%)	REPAIR $\tilde{t}$ (ms)	REPAIR f (%)	HYDRO $\tilde{t}$ (ms)	HYDRO f (%)	**total** $\tilde{t}$ (ms)
168L	6445	0.016	0.2	4.23	40.5	6.20	59.3	10
12E8	7429	0.046	0.4	3.98	39.1	6.17	60.5	10
1A4Y	8939	0.031	0.2	5.16	37.4	8.61	62.4	14
9OPY	12 388	0.037	0.2	5.73	32.7	11.7	67.1	18
1E9R	20 718	0.091	0.3	7.71	28.9	18.8	70.7	27
1A2V	33 726	0.196	0.5	10.9	27.4	28.8	72.2	40
1E9S	41 513	0.181	0.3	13.6	26.1	38.3	73.4	52
1JJ2	98 543	0.540	0.6	23.9	27.9	61.1	71.4	86
3JC8	107 640	0.495	0.2	66.4	24.6	203	75.2	270
3J79	124 514	0.371	0.3	33.1	26.4	91.8	73.1	125
3J9G	132 930	0.481	0.3	41.1	24.6	125	75.0	167
3J6B	195 137	0.839	0.7	30.6	26.6	83.6	72.7	115
1VVJ	291 123	1.30	0.5	74.1	26.3	205	72.7	282
4BTS	315 512	1.71	0.5	85.1	25.0	252	73.9	341
7EZX	944 406	6.81	0.6	281	23.9	878	74.8	1173
6QVK	1 155 500	8.30	0.5	366	23.8	1164	75.6	1539
3J3Q	2 440 800	17.8	0.5	779	23.8	2474	75.6	3270
9MJN	3 819 839	27.3	0.5	1207	23.8	3840	75.7	5074
